# The Potential Effects of Exercise Training on Cortical Glutamatergic Synapse, Retrograde Endocannabinoid Signaling, and the Oxytocin Signaling Pathway in the Diabetic–Obesity Cortex: An In Silico Study

**DOI:** 10.3390/ijms27010266

**Published:** 2025-12-26

**Authors:** Yin-Yu Chiang, Michael Anekson Widjaya, Shin-Da Lee

**Affiliations:** 1 PhD Program for Health Science and Industry, China Medical University, Taichung 406040, Taiwan; u112310801@cmu.edu.tw; 2 Graduate Institute of Biomedical Sciences, College of Medicine, China Medical University, Taichung 40402, Taiwan; 3 PhD Program in Healthcare Science, Department of Physical Therapy, China Medical University, Taichung 406040, Taiwan

**Keywords:** exercise training, Zucker (type II diabetes) rats, cortex, brain, transcriptomics, neuronal pathway, metabolic adaptation

## Abstract

Exercise training reduces metabolic dysfunction and improves neural function; however, its cortical molecular effects in diabetic–obese conditions remain unclear. Here, we aimed to identify transcriptional pathways by integrating physiological evaluation with an in silico analysis of cortical RNA-seq data from Zucker Fatty Diabetes Mellitus rats following a 12-week swimming training program. Exercise training reduced body weight and improved glucose control and blood pressure. RNA-seq analysis revealed 814 differentially expressed genes, with pathway enrichment highlighting glutamatergic synapse, retrograde endocannabinoid signaling, and oxytocin signaling pathways. These coordinated transcriptional shifts involved genes related to excitatory neurotransmission, neuromodulatory feedback, and calcium-dependent regulation. As hypothesis-generating models, these pathway-level patterns suggest that exercise training may modulate cortical signaling properties in diabetic–obese states and provide a conceptual framework for future mechanistic investigation.

## 1. Introduction

Diabetes exerts substantial stress on the brain, particularly within cortical regions involved in metabolic regulation, cognition, and sensory integration. Chronic hyperglycemia, mitochondrial dysfunction, and inflammatory signaling progressively disrupt cortical homeostasis, leading to altered neuronal excitability and transcriptional regulation [1,2,3]. These effects may be further exacerbated when diabetes coexists with obesity, as excess adiposity imposes additional endocrine and metabolic burdens that increase cortical vulnerability [4]. Accumulating evidence indicates that type 2 diabetes is associated with cortical excitability alterations, neuroinflammatory activation, and synaptic dysregulation; however, the molecular mechanisms underlying these cortical impairments remain insufficiently defined.

Exercise training is well recognized for improving systemic metabolic outcomes in diabetes and obesity, including enhanced glucose regulation, reduced inflammation, and improved cardiovascular function [5,6,7]. Beyond these peripheral effects, exercise training also enhances brain health by promoting mitochondrial capacity, neurotrophic signaling, and synaptic resilience [8,9,10]. Despite these benefits, the molecular pathways through which exercise training modulates cortical function under diabetic–obese conditions remain poorly characterized.

Transcriptomic profiling provides a powerful approach for examining such adaptations. RNA sequencing enables the detection of broad transcriptional shifts across diverse signaling networks, making it well suited for investigating complex metabolic–neuronal interactions within the cortex. Previous studies in high-fat-diet-induced obesity models have reported marked alterations in cortical synaptic, metabolic, and calcium-related gene networks [11]. In parallel, human and animal studies have demonstrated impaired cortical plasticity and reduced neuronal excitability in diabetic states [12,13], suggesting heightened vulnerability of cortical circuits. However, exercise-trained cortical transcriptomic adaptations in diabetic–obese conditions, particularly those involving neuronal signaling pathways, remain largely unexplored.

In the present study, we integrated physiological assessments, including body weight, glucose levels and systolic blood pressure, with an in silico analysis of cortical RNA-seq data from diabetic–obese rats subjected to a 12-week swimming training program. Rather than aiming to define definitive molecular mechanisms, this study sought to identify exercise training-modulated neuronal pathways associated with cortical adaptation in diabetic–obese states. Through pathway enrichment and cross-network analyses, we highlight several candidate processes, including glutamatergic synapse regulation, retrograde endocannabinoid signaling, and oxytocin-associated pathways, that may contribute to neuro-metabolic modulation to exercise training. This hypothesis-generating analysis addresses an important knowledge gap regarding how cortical neuronal signaling networks are modulated by exercise training under metabolic dysfunctions.

## 2. Results

### 2.1. Overview of Experimental Workflow and Transcriptomic Dataset

**Figure 1:**
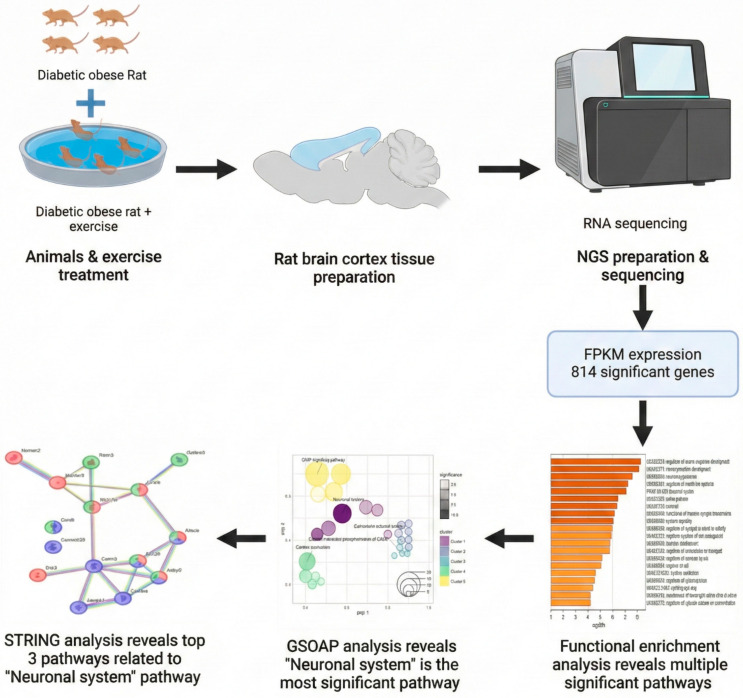
Workflow for identifying exercise-trained genes in cortex. Schematic overview of experimental design, RNA-seq analysis, and functional enrichment pipeline leading to pathway identification.

A schematic overview of the experimental design and analytical workflow, including animal grouping, exercise intervention, cortical tissue processing, RNA sequencing, and downstream bioinformatic analyses, is presented in Figure 1.

RNA sequencing analysis identified a total of 814 genes that were significantly altered by exercise training in obese diabetic rats (*p* < 0.05). These differentially expressed genes were used for subsequent functional enrichment and network-based analyses.

### 2.2. Physiological and Metabolic Adaptations After Exercise Training

Exercise training produced clear physiological and metabolic changes in obese diabetic rats. Body weight was significantly lower in the exercise-trained (EX) group compared with the obese sedentary (OB) group, accompanied by a reduction in fasting blood glucose levels (Table 1). Among blood pressure parameters, only systolic blood pressure showed a significant decrease following exercise training. These findings confirm that the 12-week swimming intervention elicited measurable metabolic and cardiovascular adaptations in ZFDM rats.

### 2.3. Identification of Neuronal-Related Gene Networks After Exercise Training

**Figure 2:**
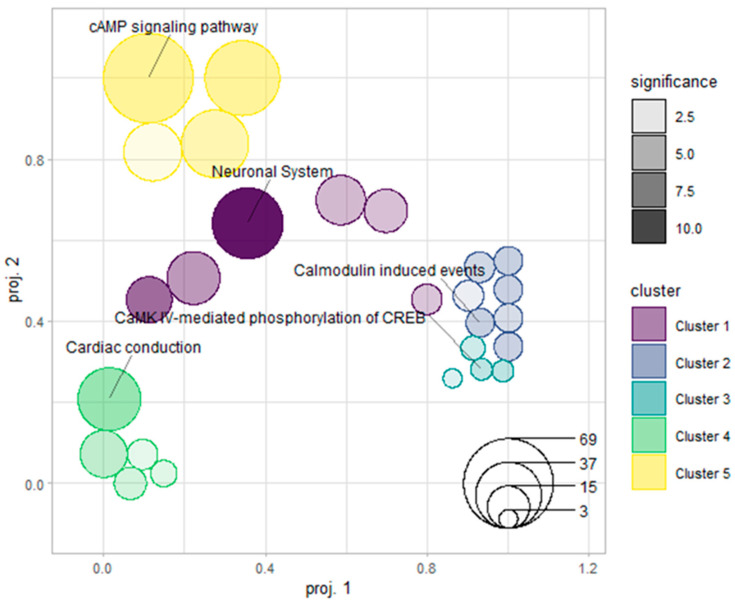
GSOAP clustering of Reactome-enriched pathways. This bubble plot displays the semantic similarity structure among enriched pathways identified from Reactome analysis. Bubble positions correspond to semantic proximity, bubble size reflects the number of genes mapped to each pathway, and grayscale shading indicates enrichment significance. Five pathway clusters were identified, with the neuronal-related cluster prominently represented in the analysis.

Functional enrichment analysis using Metascape, followed by pathway clustering with GSOAP, identified several pathway clusters after exercise training in the cerebral cortex (Figure 2). Among these clusters, one neuronal-related group comprised 44 genes associated with synaptic transmission, neuromodulation, and intracellular signaling processes.

Protein–protein interaction (PPI) analysis of these 44 genes using the STRING database revealed a densely connected network, indicating extensive molecular connectivity within this neuronal-related cluster (Figure 2). To provide full network context, a detailed STRING interaction map was generated (Supplementary Figure S1).

### 2.4. Pathway-Level Mapping of Convergent Neuronal Signaling Processes

**Figure 3:**
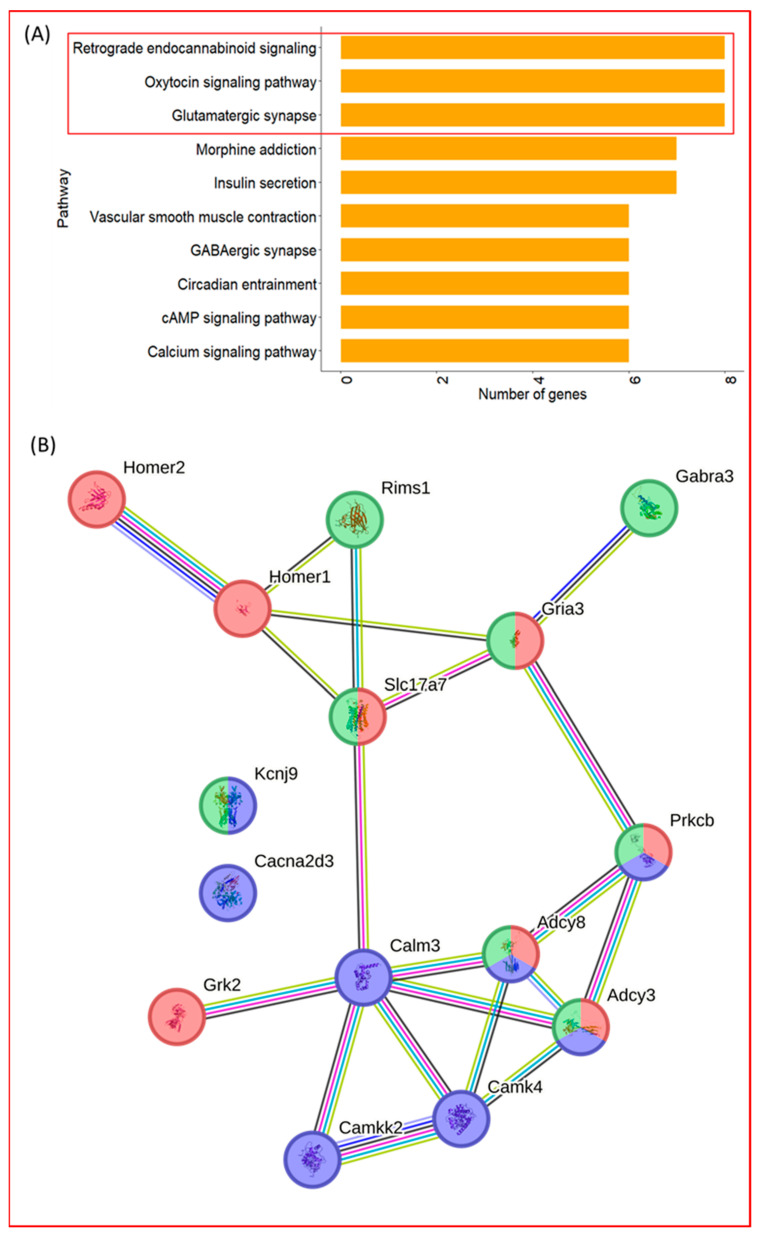
Enriched neuronal pathways and corresponding interaction structure. (**A**) Top enriched neuronal pathways derived from the 44 neuronal-related genes. Bars indicate the number of genes mapped to each KEGG pathway, highlighting glutamatergic synapse, retrograde endocannabinoid signaling, and oxytocin signaling as the most represented pathways. (**B**) STRING protein–protein interaction network of genes mapped to the three enriched pathways. Nodes represent genes and edges denote predicted functional associations. The network illustrates the overall connectivity among pathway members. Red = Glutamatergic synapse; Green = Retrograde endocannabinoid signaling; Blue = Oxytocin signaling pathway.

To determine which neuronal signaling pathways were most prominently represented within the neuronal gene set, pathway-specific mapping was performed for the 44 neuronal-related genes. Among the pathways examined, three KEGG signaling pathways contained the highest number of mapped genes: the glutamatergic synapse, oxytocin signaling, and retrograde endocannabinoid signaling (Figure 3A).

Three genes—*Adcy3*, *Adcy8*, and *Prkcb*—were shared across all three pathways, forming overlapping nodes within the pathway maps. Consistent with this overlap, STRING-based interaction analysis indicated that genes from these pathways formed an interconnected network rather than isolated modules (Figure 3B).

## 3. Discussion

Metabolic measurements confirmed that exercise training showed clear physiological improvements in obese diabetic rats. Specifically, exercise training resulted in reduced body weight, lower fasting glucose levels, and decreased systolic blood pressure. These results are consistent with previous findings showing that exercise training improves metabolic status in diabetic–obese conditions [14,15]. The presence of these expected metabolic adaptations supports the reliability of the exercise intervention and provides physiological context for interpreting the transcriptomic modulation observed in the cortex.

High-throughput RNA-seq analysis revealed substantial transcriptional changes associated with exercise training, identifying 814 differentially expressed genes. Among the pathway clusters highlighted by GSOAP, the neuronal system emerged as the most prominent. Given the cortical origin of the samples, exercise training modulation of neuron-related genes is plausible; however, the broad 44-gene cluster required further resolution to identify more interpretable biological patterns. To refine these findings, we examined the protein–protein interaction structure of the neuronal gene set using STRING. Three pathways—glutamatergic synapse, retrograde endocannabinoid signaling, and oxytocin signaling—showed the strongest representation and formed an interconnected network, unified through shared nodes such as *Adcy3*, *Adcy8*, and *Prkcb* (Figure 3B).

### 3.1. Transcriptomic Signatures Related to Glutamatergic, Excitability, and Glucose Regulatory Processes After Exercise Training

Across the three neuronal-related pathways, *Adcy3*, *Adcy8*, and *Prkcb* were consistently present, serving as shared nodes that linked multiple signaling processes within the exercise training-modulated network (Figure 3B). Additional cross-pathway overlaps, including *Slc17a7*, *Gria3*, and *Kcnj9*, further illustrated the interconnected nature of excitatory, retrograde, and neuromodulatory gene groups. These patterns indicate that exercise training may influence network-level organization rather than individual pathways in isolation.

Interpretation of these relationships must remain cautious, as transcriptomic data do not establish functional or mechanistic consequences. Nonetheless, the observed pathway signatures are broadly consistent with neural adaptations reported in metabolic or exercise-related contexts. For example, glutamatergic dysregulation has been described in diabetic cortical conditions, supporting the relevance of excitatory signaling within this network [16]. Exercise training has also been shown to enhance endocannabinoid tone in metabolic and neural settings, further aligning with the observed enrichment of endocannabinoid-related genes [17]. In addition, studies in diabetic neuropathic pain models demonstrate that exercise training can attenuate neuroinflammatory and excitability-related disturbances [18], suggesting that multiple neuronal conditions in diabetes-associated environments may be improved after exercise training.

**Figure 4:**
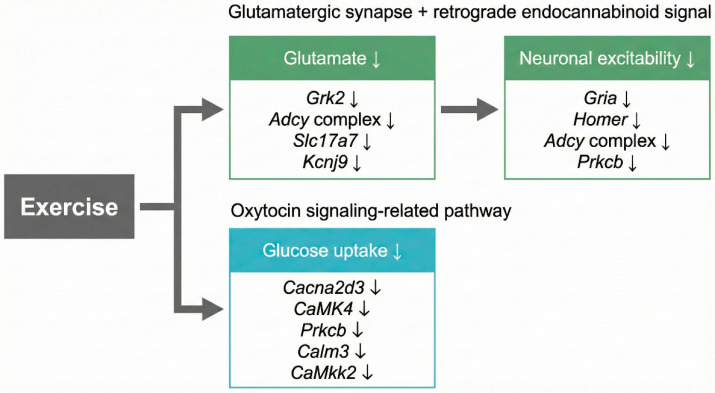
Hypothetical model integrating glutamatergic synapse, retrograde endocannabinoid signaling, and oxytocin signaling pathway based on KEGG pathway annotations and STRING interaction analysis.

Altogether, these observations prompted further consideration of how exercise training-associated transcriptional changes may converge across multiple neuronal signaling pathways in the diabetic–obese cortex (Figure 4). Exercise training potentially reduced glutamate through *Grk2*, *Adcy* complex, *Slc17a7*, and *Kcnj9*. Reduced glutamate levels may contribute to decreased neuronal excitability, potentially through downregulation of *Gria*, *Homer*, *Adcy* complex, and *Prkcb* gene expression. Exercise training also affected the oxytocin signaling-related pathway which led to lower glucose uptake. Glucose uptake was reduced during exercise due to lower gene expression of *Cacna2d3*, *CaMK4*, *Prkcb*, *Calm3*, and *CaMkk2*. While the present findings remain hypothesis-generating and require future mechanistic validation, the convergence of glutamatergic, endocannabinoid, and oxytocin signaling pathways identifies several molecular candidates that warrant further investigation. These pathway-level insights may guide subsequent studies aiming to clarify how exercise influences cortical neuronal regulation under diabetic–obese conditions.

### 3.2. Integration of Physiological and Cortical Transcriptional Adaptations

Exercise training produced clear metabolic improvements, including reductions in body weight and fasting glucose and lower systolic blood pressure. These physiological changes are consistent with previous evidence showing that exercise training enhances metabolic status in diabetic–obese conditions [4,14,15]. Such systemic adaptations may coincide with transcriptional changes observed in the cortex, although direct relationships were not assessed in the present study.

Within the cortical transcriptome, exercise training was associated with shifts involving glutamatergic synaptic signaling, retrograde endocannabinoid pathways, and oxytocin-related neuromodulatory processes. Although these associations do not establish causality, the direction of several transcriptional patterns is observed alongside neural adaptations previously described in diabetic or exercise contexts [16,17].

Taken together, these observations suggest that systemic benefits induced by exercise training may coincide with cortical signaling adjustments, providing a conceptual framework for understanding how exercise training could support neuronal adaptation under diabetic–obese conditions. However, these inferences remain hypothesis-generating, and future mechanistic studies will be required to determine whether such transcriptomic shifts have functional consequences.

### 3.3. Limitations

Several limitations should be acknowledged. First, the transcriptomic analysis was based on four biological replicates per group, which is common in exploratory RNA-seq studies but may limit the detection of subtle expression changes. Second, only male rats were included, restricting generalizability to females, who may exhibit distinct neuro-metabolic modulation to exercise training. Third, swimming exercise training may introduce stress-related transcriptional effects, and individual variability in exercise adaptation was not assessed. Fourth, the study relied exclusively on mRNA profiles without protein-level or functional assays, and pathway interpretation should therefore be considered hypothesis-generating rather than mechanistic. Future work incorporating region-specific assays, multimodal omics, and functional manipulation will be necessary to validate the cortical pathways identified here.

Despite these limitations, the analysis highlighted three neuron-related pathways—glutamatergic synapse, retrograde endocannabinoid signaling, and oxytocin signaling—that showed coordinated transcriptional patterns following exercise training. These findings provide a conceptual framework for exploring how exercise training may influence cortical signaling in diabetic–obese conditions. However, these interpretations remain speculative, and additional mechanistic studies will be required to determine whether the observed transcriptomic patterns translate to functional outcomes. Accordingly, these findings should be interpreted as hypothesis-generating and require future functional validation.

## 4. Materials and Methods

### 4.1. Animals

Male Obese Zucker Diabetic rats were used as a polygenic model of type 2 diabetes with obesity. Lean and obese Zucker rats were divided into three groups, sedentary Lean Zucker rats (LN), sedentary Obese Zucker Diabetic rats (OB), and Obese Zucker Diabetic rats after exercise training (OB-EX), with 10 rats per group. For RNA sequencing, four of the ten animals in each group were selected to represent transcriptomic profiles. All rats were housed under controlled temperature and lighting conditions with ad libitum access to standard chow and water.

### 4.2. Physiology and Metabolic Analysis

Systolic, diastolic, and mean arterial blood pressures were measured using an automated tail-cuff system (29SSP; IITC/Life Science Instruments, Woodland Hills, CA, USA), following procedures previously established in our laboratory for obese rat exercise studies. Rats were habituated to the apparatus for several sessions prior to data collection to minimize stress-induced variability, and at least three consecutive stable readings were averaged for each parameter.

Fasting blood glucose concentrations were obtained from tail vein blood samples using Roche Accu Soft test strips, based on protocols applied in earlier investigations of exercise training in obese or diabetic rats [19]. All glucose measurements were collected after a 10–12 h overnight fast and prior to euthanasia.

Citrate synthase (CS) activity was analyzed by homogenizing soleus tissue in HES buffer and measuring enzymatic activity spectrophotometrically according to established methods from our previous studies. Soleus CS activity is presented as μmol/min/g tissue and served as an index of oxidative enzyme capacity after exercise training.

### 4.3. Exercise Training Protocol

The Obese Zucker Diabetic rats after exercise training (OB-EX) group underwent a swimming training program modified from earlier studies conducted in obese diabetic rats [19]. Rats swam for 15 min per day, 5 days per week during the first 2 weeks. Training duration was increased to 20 min per day in week 3 and then maintained at 30 min per day from weeks 4 to 12. All exercise sessions were performed in a 60 × 90 × 50 cm tank filled to an appropriate depth and maintained at 35 °C to prevent hypothermia.

To minimize handling stress, animals were gently transferred into and out of the swimming tank and monitored continuously throughout each session. After swimming, rats were thoroughly dried using towels followed by a warm air dryer. All animals were euthanized 48 h after the final training session to avoid acute exercise effects on physiological or molecular measurements.

### 4.4. RNA Isolation

Total RNA was extracted from the cerebral cortex using a standard phenol–chloroform procedure based on the manufacturer’s guidelines and our laboratory’s established protocol for neural tissue processing. Approximately 30 mg of frozen cortical tissue was homogenized in 1 mL of TRIzol reagent (Invitrogen, Carlsbad, CA, USA) using a motorized homogenizer, followed by phase separation with chloroform and centrifugation at 12,000× *g* for 15 min at 4 °C. The aqueous phase was transferred to a fresh tube, and RNA was precipitated with isopropanol, washed with 75% ethanol, and resuspended in RNase-free water.

RNA concentration and purity were assessed by spectrophotometric measurement (NanoDrop 2000; Thermo Fisher Scientific, Waltham, MA, USA). Samples with A260/280 ratios between 1.9 and 2.1 were retained for sequencing. RNA integrity was further evaluated using microfluidic electrophoresis (Bioanalyzer 2100; Agilent Technologies, Santa Clara, CA, USA), and only samples with an RNA integrity number (RIN) ≥ 7.0 were included in downstream library preparation. RNA samples were stored at −80 °C until sequencing.

RNA-seq library preparation and sequencing were performed by Genomics, BioSci & Tech Co. (New Taipei City, Taiwan) using a standard TruSeq-based protocol on an Illumina NovaSeq platform (150 bp paired-end).

### 4.5. RNA-Seq Data Analysis and Functional Enrichment

Differential gene expression was defined based on FPKM-derived expression values using a significance threshold of *p* < 0.05 without a fold-change cutoff, consistent with exploratory transcriptomic analyses. Functional enrichment analysis was performed using Metascape (https://metascape.org, accessed on 21 December 2023) with default settings (minimum overlap = 3, *p* < 0.01) [20]. Pathway clustering was conducted using GSOAP (https://gsoap.csb.pitt.edu, accessed on 21 December 2023) based on gene overlap-derived similarity between pathways [21]. Protein–protein interaction networks were generated using the STRING (version 11.5) database with a medium-confidence interaction threshold (interaction score ≥ 0.7) [22]. These analytical parameters are reported to ensure transparency and reproducibility, as requested by reviewer comments.

### 4.6. Additional Software and Visualization

Visualization of gene expression patterns and network structures was performed using the ggpubr package (https://cran.r-project.org/package=ggpubr, accessed on 21 December 2023) and related R-based tools [23]. These analyses supported pathway-level interpretation of transcriptional changes as hypothesis-generating in silico findings. BioRender (https://www.biorender.com, accessed on 21 December 2023) was used to generate the schematic illustration shown in Figure 1.

### 4.7. Ethics Statement

All animal experimental procedures were reviewed and approved by the Institutional Animal Care and Use Committee (IACUC) of China Medical University, Taichung, Taiwan (Approval No. CMUIACUC-104-183-N), approved on 30 December 2014. All procedures complied with institutional and national guidelines for the care and use of laboratory animals.

## 5. Conclusions

Exercise training produced clear metabolic improvements in obese diabetic rats, including reductions in fasting glucose and systolic blood pressure. In parallel with these physiological effects, transcriptomic analysis revealed broad exercise-associated changes in cortical gene expression. Pathway enrichment consistently highlighted three neuron-related pathways—glutamatergic synapse, retrograde endocannabinoid signaling, and oxytocin signaling—as prominent components of the cortical response to exercise. The shared and interconnected nature of these pathways indicates that exercise is associated with coordinated, network-level transcriptional patterns rather than changes confined to isolated signaling routes.

Although RNA-seq data cannot determine directionality or functional consequences, several observed patterns aligned with gene groups involved in excitatory, neuromodulatory, and Ca^2+^-related signaling processes. These observations should be interpreted as hypothesis-generating, as no functional or correlational analyses were performed to establish mechanistic relationships. Accordingly, future studies will be required to clarify the functional relevance of these pathways in the diabetic–obese cortex.

Overall, this work provides a conceptual framework suggesting that exercise may reshape cortical signaling networks under metabolic dysfunctions. The findings offer a foundation for future investigations aimed at defining how exercise influences neuronal regulatory processes in diabetic–obese conditions.

## Figures and Tables

**Table 1:** Metabolic and Cardiovascular Parameters.

Parameter	LN (*n* = 10)	OB (*n* = 10)	OB-EX (*n* = 10)
Body Weight (g)	372 ± 40	456 ± 27 **	406 ± 45 ^#^
Glucose (mmol/L)	4.8 ± 0.4	22.4 ± 2.5 **	13.4 ± 4.5 *^#^
SBP (mmHg)	121 ± 9	135 ± 8 *	124 ± 7 ^#^
DBP (mmHg)	83 ± 10	90 ± 11	87 ± 14
MBP (mmHg)	96 ± 10	105 ± 10	99 ± 11
CS Activity (μmol/min/g)	29.1 ± 6.9	32.5 ± 7.3	59.9 ± 8.2 **^##^

LN: sedentary Lean Zucker rats. OB: sedentary Obese Zucker Diabetic rats. OB-EX: Obese Zucker Diabetic rats after exercise training. Values are means ± SD. BW, body weight; SBP, systolic blood pressure; DBP, diastolic blood pressure; MBP, mean blood pressure; CS, citrate synthase activity in Soleus. * *p* < 0.05, ** *p* < 0.01 indicate significant differences from LN. ^#^ *p* < 0.05, ^##^ *p* < 0.01 indicate significant differences between OB and OB-EX.

## Data Availability

The data presented in this study are available on request from the corresponding author. (Raw sequencing files are available upon reasonable request due to ongoing related research activities).

## Supplementary Materials

The following supporting information can be downloaded at: https://www.mdpi.com/article/10.3390/ijms27010266/s1.
