# Supplementary material for: The Potential Effects of Exercise Training on Cortical Glutamatergic Synapse, Retrograde Endocannabinoid Signaling, and the Oxytocin Signaling Pathway in the Diabetic–Obesity Cortex: An In Silico Study
Source: Int J Mol Sci. 2025 Dec 26;27(1):266. doi: 10.3390/ijms27010266 (PMC12786004; doi:10.3390/ijms27010266)
Supplement: Supplementary file 1 [file ijms-27-00266-s001.zip › ijms-4039709-supplementary.pdf]

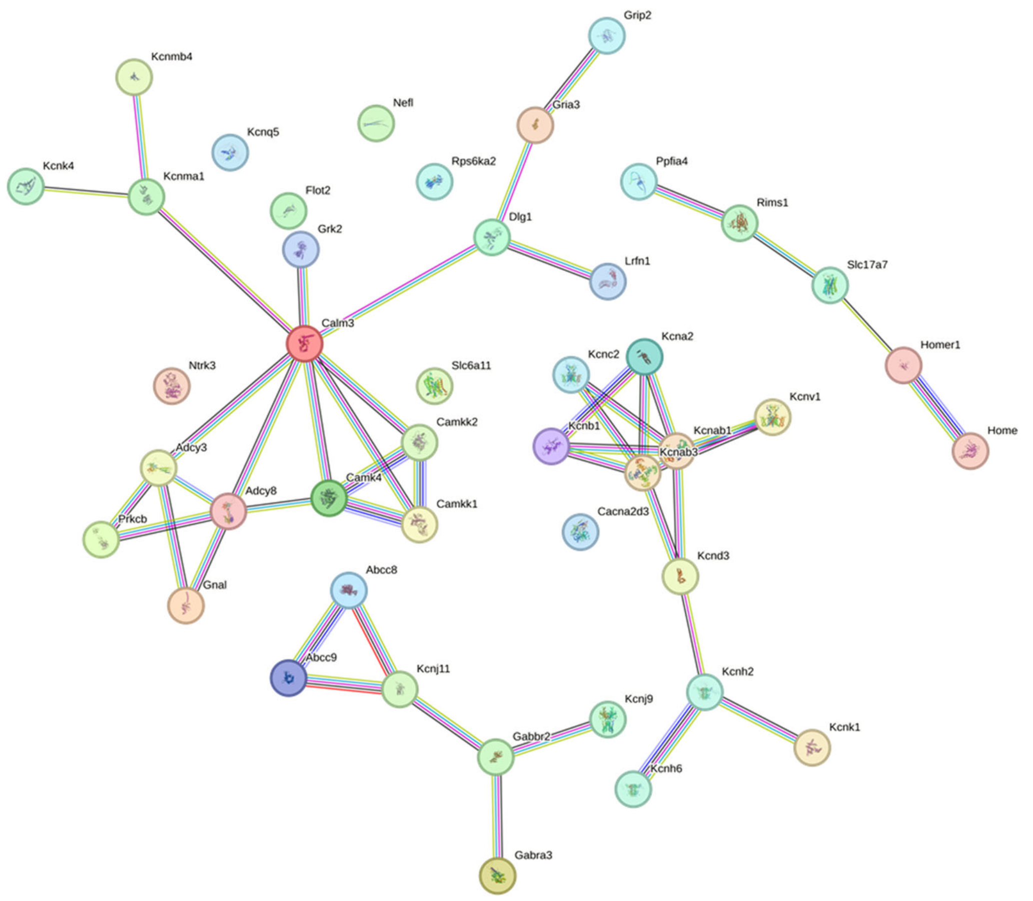

.

Supplementary Figure S1. Full STRING protein–protein interaction (PPI) network of the 44 neuronal-related genes identified from GSOAP clustering.

This figure presents the complete protein–protein interaction (PPI) network generated using the STRING database (version 12.0) for all 44 neuronal-related genes identified from the GSOAP semantic clustering analysis. The network was constructed using the STRING default high-confidence interaction score threshold (≥0.7). Each node represents a gene product, and edges denote predicted functional associations based on evidence from co-expression, experimental data, curated databases, and text-mining sources. This full-network visualization complements the simplified interaction maps shown in Figures 2 and 3 by depicting the broader connectivity structure across all neuronal-related genes in the dataset without pathway-level filtering. No mechanistic interpretations are implied, and the figure is provided solely to illustrate the comprehensive interaction landscape of the neuronal gene cluster.
